# Identification of Socio-Economic Impacts as the Main Drivers of Carbon Stocks in China’s Tropical Rainforests: Implications for REDD+

**DOI:** 10.3390/ijerph192214891

**Published:** 2022-11-12

**Authors:** Guifang Liu, Jie Li, Liang Ren, Heli Lu, Jingcao Wang, Yaxing Zhang, Cheng Zhang, Chuanrong Zhang

**Affiliations:** 1 College of Geography and Environmental Science/Key Research Institute of Yellow River Civilization and Sustainable Development & Collaborative Innovation Center on Yellow River Civilization of Henan Province, Henan University, Kaifeng 475004, China; 2 Key Laboratory of Geospatial Technology for the Middle and Lower Yellow River Regions (Henan University), Ministry of Education/National Demonstration Center for Environment and Planning, Henan University, Kaifeng 475004, China; 3 Henan Key Laboratory of Earth System Observation and Modeling, Henan University, Kaifeng 475004, China; 4 Henan Dabieshan National Field Observation and Research Station of Forest Ecosystem, Henan University, Kaifeng 475004, China; 5 Department of Geography & Center for Environmental Sciences and Engineering, University of Connecticut, Storrs, CT 06269-4148, USA

**Keywords:** carbon stocks, forest changes, REDD+, socio-economic drivers, effectiveness

## Abstract

Active incentives or compensation measures plus conservation, sustainable management of forests, and enhancement of forest carbon stocks (denoted together as “REDD+”) should be adopted in developing countries to reduce the greenhouse gas emissions caused by deforestation and forest degradation. Identification and analysis of the driving forces behind carbon stocks are crucial for the implementation of REDD+. In this study, the principal component model and the stepwise linear regression model were used to analyze the social and economic driving forces of stocks in three important types of forest change: deforestation, forestland transformation, and forest degradation in China’s tropical rainforests of Xishuangbanna, based on the combination of satellite imagery and the normalized difference vegetation index. The findings show that there are different key driving forces that lead to carbon stock changes in the forest land conversion of Xishuangbanna. In particular, the agricultural development level is the main cause of emissions from deforestation, whereas poor performance of protection policies is the main cause of emissions from forest degradation. In contrast, the rural economic development interventions are significantly positive for emissions from forestland transformation. It is crucial to pay attention to distinguishing the driving forces behind carbon stock changes from forest degradation, deforestation, and transformation for optimizing REDD+ implementation and ensuring the effectiveness of REDD+.

## 1. Introduction

Currently, climate change is one of the most important topics of global concern. In addition to the natural causes, climate change is also caused by the effects of increased greenhouse gas emissions, such as carbon dioxide, due to anthropogenic activities [1,2,3,4,5,6]. Tropical rain forests play a key role in coping with the increase in global carbon dioxide levels [7] because tropical rainforest vegetation contains more carbon than the mid-temperate zone and frigid zone forests. At present, the carbon released by tropical rain forests due to deforestation accounts for 6–15% of the annual global greenhouse gas emissions [8,9,10,11]. However, the Kyoto Protocol, which aims to stabilize greenhouse gas concentrations and achieve global carbon emissions reductions, does not cover deforestation [12,13,14] due to verification and monitoring issues as well as factors related to additionality and permanence.

The United Nations Climate Change Conference (UNFCCC) in Copenhagen in 2009 agreed upon a program focused on reducing emissions from deforestation and forest degradation plus conservation, sustainable management of forests, and enhancement of forest carbon stocks (denoted together as “REDD+”) in developing countries through positive incentives, which could help countries to effectively respond to global climate change by enhancing forests’ ability for carbon storage [15,16,17,18]. Most recently, developing country parties implementing REDD+ activities are encouraged to complete the Warsaw Framework for REDD+, which establishes the requirements for obtaining recognition of mitigation results and for receiving the associated results-based payments, with online safeguard information systems and submissions of summaries of information to the UNFCCC. The goal of REDD+ is to raise funds from developed countries to help developing countries reduce greenhouse gas emissions caused by deforestation. Its core principle involves using market mechanisms to encourage reduction of the greenhouse gas emissions caused by deforestation by reducing forest damage and preventing forest degradation while allowing these countries to obtain corresponding income through the carbon market. Thus, the key to the implementation of REDD+ lies in the close cooperation and active participation among international organizations, countries, local governments, local residents, investors, and carbon traders [19,20,21,22,23,24]. Although the details of the REDD+ mechanism have not been finalized, some basic issues have been agreed upon, including deforestation and forest degradation monitoring report and verification, determination of the carbon emission reference levels (baselines), compensation standards for opportunity costs, compensation and financial incentives, and ecological and social benefits brought about by REDD+.

Nowadays, the forests in China are in the fourth place in terms of forest resources all over the world, which covers 20.6 percent of China’s landmass. Largely due to the government’s approach of administrative fiat and compulsory land-use zoning, the Chinese government sharply curtailed commercial timber harvesting in western and northern provinces. China’s forest cover had increased by approximately 40 million hectares since the late 1970s by 1998 (with the so-called “logging ban”) [25]. In 2003, the State Forest Administration began to encourage provinces to experiment with tenure reform and thus provide an explicit national-level policy framework. More recently, China’s newly revised Forest Law introduced greater change when it came into force in July 2020. Shifting away from a focus on timber production, the revised law seeks to balance forest management to more fully realize the role of forests in providing economic, social, ecological, and cultural services.

Forest change is a special natural and social phenomenon brought about by changes to natural attributes and human utilization [26,27,28,29,30]. The natural driving forces generally relate to the background conditions of forest changes; as no significant changes in this category occur in a short time, the impact on forest changes is small. Social and economic driving forces are the most fundamental driving forces for forest utilization change and the evolution of related carbon stocks. Forest conversion for cattle ranching in the Amazon has led to around 17% forest loss in populated areas, roads, and rivers for 50 years [31,32]. In recent years, mahogany, gold, and oil are additional drivers for deforestation and forest degradation in remote areas of this region [33]. About half of the illegal removal of timber from forests in East Africa is largely due to fuel wood harvesting [34,35]. In addition, most of Indonesia’s deforestation is driven by expanding agriculture such as rice, rubber, and palm oil [36,37,38,39]. The identification and analysis of the socio-economic forces driving forest change form the basis for the implementation of REDD+ projects [40,41,42,43].

Xishangbanna has China’s most complete, most typical, and largest tropical rain forest ecosystem, which comprises 16 percent of China’s total plant diversity [44]. Deforestation in Xishuangbanna emitted almost 90 million tons of carbon stocks for the period 1976–2003 and will lead to a further 4 million tons of carbon emissions under current deforestation rate in the next 20 years [45,46,47]. Such forestland conversion will also cause significant loss of biodiversity, substantial soil erosion, and large declines in other ecosystem services, including the production of clean water, water conservation, and the supply of non-timber forest products [48]. As a result, it is important to balance economic growth and conservation goals for local policy makers in this rapidly developing region.

Due to geographical conditions and economic demands, rubber plantations have greatly been developed since the 1990s. The large area of tropical rain forest and tropical monsoon rain forest in Xishuangbanna play a strong role in the formation and retention of fog and thus alleviates the lack of rainfall during dry season. Such geographical conditions are conducive to the growth of rubber plantations and various other plantations [49]. On the other hand, the local government believes that rubber plantations could not only meet the needs of China’s rapidly industrialized demand but also increase the household income of farmers. As a result, farmers were encouraged to plant rubber on steep slopes in the 1980s. In the 1990s, the state subsidized the rubber price for farmers as a very stable income. In the early years of the 21st century, farmers further expanded rubber plantations under the stimulus of the state [50]. Due to the soaring market price in 2006, the rubber plantation area in Xishuangbanna reached the limit of 3 million mu [51]. Similar policies also happened with other plantations in Xishuangbanna, especially tea plantations. Since the 11th Five-Year Plan, governments at all levels have invested a lot of financial, material, and human resources in tea plantations [52,53].

Prior studies have generally been conducted in tropical forests in Sub-Saharan Africa, Pacific Asia, or Latin America and the Caribbean. However, there are only a few studies where the Xishuangbanna tropical rainforest region in Southwest China forms the focus. With the increasing regional population pressure, the economically important plantations expansion (especially rubber plantations and tea plantations) and the economic growth, the carbon stocks of Xishuangbanna reduced significantly, and to provide practical REDD+ suggestions for the local policy makers through distinguishing the driving forces behind carbon stock changes from forest degradation, deforestation, and transformation is the main contribution in this paper. This will benefit optimizing REDD+ implementation and ensuring the effectiveness of REDD+ in China’s tropical rainforests.

Thus, taking the Xishuangbanna region in China as the research subject, this paper presents a comprehensive analysis of the driving forces of carbon stocks.

The objectives of the study are as follows:

● Extract land use change information for the period 1992 to 2007 as a baseline for REDD+ program in Xishuangbanna.
● Describe the social and economic driving forces for emissions from land use changes, with a particular focus on deforestation, forestland transformation, and forest degradation.
● Identify the roles of socio-economic development, agricultural development level, and policies in causing carbon stock changes from deforestation, forestland transformation, and forest degradation.
● Provide a description of the pathway to conservation of existing forests carbon stocks in the context of REDD+.

## 2. Materials and Methods

### 2.1. Study Area

The Xishuangbanna region is home to the majority of tropical forest ecosystems in China. The topography, climate, and soil of Xishuangbanna are suitable for the growth and reproduction of various organisms. Moreover, 4500 species of higher plants have been recorded in Xishuangbanna, accounting for about one-seventh of the total number of higher plants in China. The native vegetation types include those found in tropical rain forests, montane rain forests, tropical monsoon forests, subtropical evergreen broad-leaved forests, deciduous broad-leaved forests, warm coniferous forests, and bamboo forests as well as shrubs and grasses [54,55,56,57]. The species of tropical rain forests are *Parashorea chinensis*, *Canarium Bengalese* et al., and the dominant tree species of montane rain forests are *Alstonia scholaris*, *Paramichelia baillonii* et al. [58,59]. Frequent tree species of tropical monsoon forests are *Bombax ceiba*, *Ficus altissima* et al. [60,61,62]. The subtropical evergreen broad-leaved forests have two conspicuous tree layers: the top layer, 15–25 m tall, and the lower layer, 3–15 m tall. The top layer is dominated by species in the *Fagaceae* and *Lauraceae* and the frequent species in the lower layer are *Syzygium tetragonum*, *Tricalysia fruticose* et al. [63]. In recent years, due to the increase in the population, intensification of anthropogenic activities, the enabling climate, and suitable terrain conditions in the area, the cultivation of rubber, oil palm, and tea has risen rapidly. Thus, the changes in forestland have been very dramatic.

### 2.2. Data Sources

#### 2.2.1. The Procedure Scheme and Interpretation of Land Use Change

This study divides the land use cover in Xishuangbanna region into nine types: forestland, shrub, dry land, rubber plantations, paddy fields, wild grassland, construction land, tea plantations, and other land. We mainly analyze three types of forest change, namely deforestation, forestland transformation, and forest degradation. Deforestation refers to the conversion of forestland to dry land, paddy fields, wild grass, construction land, and other land (facility agricultural land, ridges, saline land, sandy land, bare land, etc.). Forestland transformation refers to the conversion of forestland to rubber plantations and tea plantations, and forest degradation refers to the conversion of forestland to shrubs.

Under the REDD+ financial compensation framework, the baseline could be used to assess how much emissions reduction would be achieved for compensation via REDD+ implementation compared with the non-implementation scenario. Historical deforestation periods are often used as REDD+ baselines to define the emissions intensity and scale. The period of 1992–2007 was the most serious deforestation period in Xishuangbanna. Therefore, the land use changes for the period were set as the REDD+ baseline to evaluate the potential carbon emissions reduction in this area.

**Figure 1:**
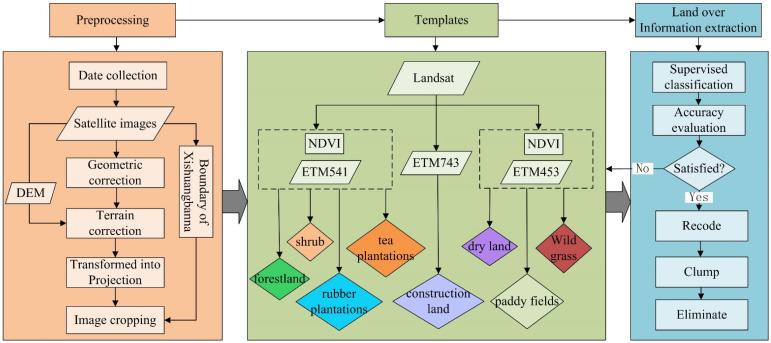
Image processing procedure.

Due to availability, accessibility, and quality of Landsat data, satellite images of the study region from 1992, 1999, 2003, and 2007 were used to obtain information on land use changes (Figure 1). The remote sensing images were sourced from the Landsat7 ETM, Landsat5 TM, and Terra MODIS NDVI (MOD13Q1) data provided by the Geospatial Data Cloud (http://www.gscloud.cn, (accessed on 1 October 2022)) of the Computer Network Information Center of the Chinese Academy of Sciences. The spatial resolution of all the Enhanced Thematic Mapper/Thematic Mapper (ETM/TM) images was 30 m, and the product type was L1T (Level 1T standard terrain correction). System radiation and ground control point geometric corrections were performed, and the terrain correction was conducted using the digital elevation model. The UTM-WGS 84 Antarctica Polar Projection was used as the map projection. The ETM+ data in 2007 were repaired using the strip repair model provided by the Geospatial Data Cloud (http://www.gscloud.cn, (accessed on 1 October 2022)). Multi-image adaptive local regression was used as the repair method.

The downloaded remote sensing images were transformed into projections and converted to the Albers equal area cut cone standard projections for more accurate calculations of the areas. The grid analysis tool was used to mosaic the image data of the same year, following which the administrative boundary of Xishuangbanna was applied as a mask to crop the image of the Xishuangbanna area.

The terrain of the study area is more complex, and the land cover types are diverse. Since NDVI quantifies vegetation by measuring the difference between near-infrared and red light, it is useful in understanding vegetation density for forests, plantations, and grass to assess changes in land use. As such, the forest areas were separated in the study using combinations of the ETM + 541 band and the NDVI data. Combining the ETM + 453 band with the NDVI data helps extract the cultivated land information, while using the ETM + 743 band can extract the information on the construction land. Preliminary classification results were obtained based on the selection of the training samples (i.e., area data that are considered representative of each land use type to be classified) for supervised classification, and accuracy testing was performed. If the results did not match, the training samples were selected once more, and supervised classification and precision testing were performed in a loop until the minimum error was obtained. Finally, the classification results were recoded, clumped, and eliminated to remove broken patches with an area of less than 1 ha (10,000 m^2^) and to unite the smallest unit.

#### 2.2.2. Socio-Economic Driving Indicators

Among socio-economic driving forces, population, economic development, living standards, agricultural development level, and agricultural technological progress are the main forces affecting land use changes. Population, which was about 1.065 million in Xishuangbanna in 2007, is the most important force and one of the most dynamic forces. Economic development is the fundamental force since the development of the second and third industries and the market-oriented allocation of resources increases the demand for land. Through the diffusion of lifestyles and consuming values, the growth in the living standard affects land resources redistribution. It is likely that agricultural development level and agricultural technological progress will directly lead to a significant change of the land uses as small subsistence farms are transformed into larger units. Therefore, 26 driving factors fall into five categories through combining the information on the socio-economic development of Xishuangbanna from Yunnan Statistical Yearbook [64] and the forest changes analyzed above (Table 1). Data sources of Yunnan Statistical Yearbook are obtained from annual statistical reports of government, containing the following 18 parts: (1) Survey; (2) National Account; (3) Investment in Fixed Assets; (4) Urban and Rural Consumption; (5) Public Finance; (6) Foreign Trade; (7) Agriculture and Country; (8) Industry and Energy; (9) Construction and Real Estate; (10) Transport, Communications and Service Industry; (11) Banking and Insurance; (12) Tourism; (13) Education, Science, Technology and Culture; (14) Public Health, Sports and Social Services; (15) Population and Employment; (16) Resources and Environment; (17) Survey of National Autonomous Area; (18) Survey of Intra-county Economies.

### 2.3. Research Method

**Figure 2:**
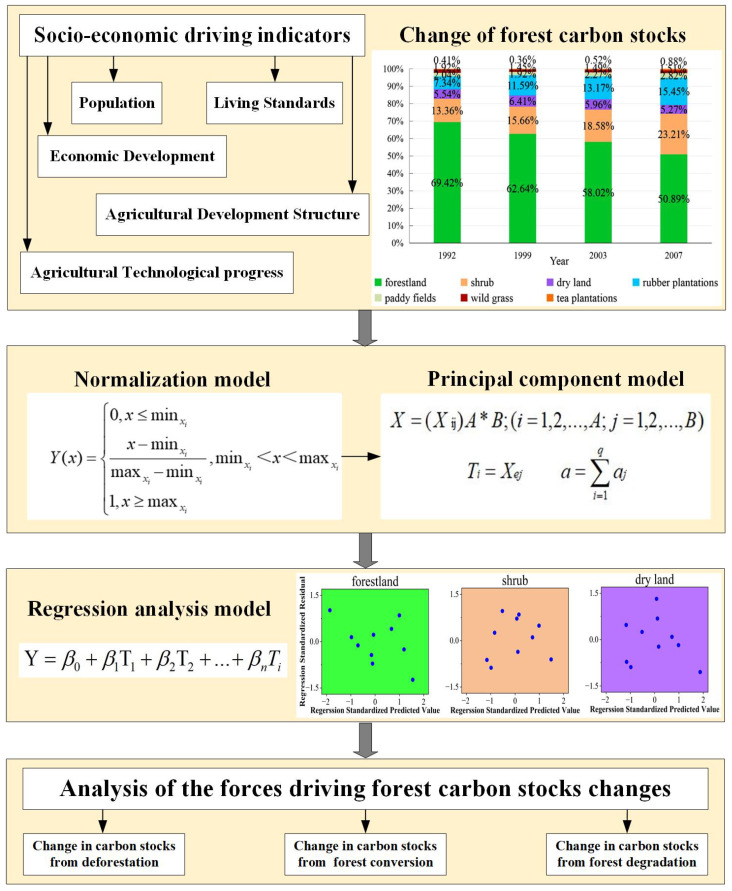
Methodological framework.

The first normalization model was used for the standardization. Then principal component analysis was utilized to retain the main information. Finally, the stepwise linear regression model was adopted to establish the functions for analyzing drivers of carbon stock changes from deforestation, forestland transformation, and forest degradation in Xishuangbanna. Figure 2 is the overall methodological framework for the study.

#### 2.3.1. IPCC Greenhouse Gas Inventory Method

According to IPCC Guidelines on Good Practices in Land Use, Land Use Change and Forestry [65], the carbon pool includes three sub-pools: live biomass (LB), dead organic matter (DOM), and soil organic matter (SOM). Among them, LB includes aboveground biomass (AB) and underground biomass (BB) and DOM includes dead wood (DW) and litter (LT).

The total carbon pool can be expressed as:

C_Total_ = C_LB_ + C_DOM_ + C_SOM_ (1)

where C_Total_ is the total carbon pool of land ecosystem; C_LB_ is live biomass carbon pool; C_DOM_ is dead organic matter carbon pool; C_SOM_ is soil organic carbon pool.

Among them:

C_DOM_ = C_DW_ + C_LT_ (2)

where C_DW_ is dead wood carbon pool; C_LT_ is litter carbon pool.

Carbon stocks assessment of land use change refers to calculating the changes of LB, DOM, and SOM:

ΔC_Total_ = ΔC_LB_ + ΔC_DOM_ + ΔC_SOM_ (3)

here, ΔC_Total_ is the change of total carbon pool (t/a), ΔC_LB_ is the change of carbon pool in LB (t/a), ΔC_DOM_ is the change of carbon pool in DOM (t/a), and ΔC_SOM_ is the change of carbon pool in SOM (t/a).

The change of carbon pool in DOM is the sum of the change of DW and LT:

ΔC_DOM_ = ΔC_DW_ + ΔC_LT_ (4)

The IPCC inventory method needs to balance the cost and the accuracy of the measurement. For this reason, IPCC has proposed three tiers in terms of measurement methods, parameters, and data sources. For the REDD+ mechanism, it is necessary to allow certain uncertainty in the measurement results so as to reduce costs. Researchers have conducted a lot of work on the carbon cycle of terrestrial ecosystems (such as vegetation carbon density, soil carbon storage, and so on) in Xishuangbanna. For example, Zhang Xiuyu et al. studied the carbon storage of terrestrial vegetation and Li Hongmei et al. studied the carbon storage and density of soil. In addition, Xiao Ziwei et al. and Pang Jiaping studied the carbon density of vegetation and soil in tea plantation and rubber. Their research provides an important reference for the local carbon parameters (Table 2).

#### 2.3.2. Normalization Model

Due to the differences in the dimensions of the indexes, obvious differences arise in the orders of the magnitudes of the indexes. Thus, it is necessary to standardize the indicators in the index system. This study adopted the normalized method for the standardization, the formula being:

(5) $$\begin{array}{l} Y(x)=\left\{\begin{array}{l} 0,x\leq \min_{x_{i}}\\ \frac{x-\min_{x_{i}}}{\max_{x_{i}}-\min_{x_{i}}},\min_{x_{i}}<x<\max_{x_{i}}\\ 1,x\geq \max_{x_{i}} \end{array}\right. \end{array}$$

where *Y*(*x*) is the standardized index data, *x_i_* denotes the original data, and max *x_i_* and min *x_i_*, respectively, represent the maximum and minimum values of index *i* in the original sample data.

#### 2.3.3. Principal Component Model

The following principal component analysis steps were used [72,73]. Suppose A research areas and B original samples. Matrix X selects the indicators as follows:

(6) $$X=\left(X_{ij}\right)A*B$$

where (*i* = 1, 2, …, *A*; *j* = 1, 2, …, *B*).

Calculate the correlation coefficient matrix Rb*b between each index, its eigenvalues (∧1≥∧b≥0) and normalized eigenvector *ej*, thus obtaining the principal component *T_i_*.

(7) $$T_{i}=X_{ej}$$

Jolliffe et al. [74] indicated that the cut-off of cumulative 70% variation is common to retain the principal components (PCs) for analysis. The higher value, which provides a good approximation of the variation present in the original dataset, was used in this study. If the variance contribution rate of the j-th principal component is 85–95%, consider the first q principal components *T*_1_, *T*_2_, …, *T*_q_. Then, this principal component q can be used to reflect the original index information of B. The contribution rate formula is:

(8) $$a=\sum_{i=1}^{q}a_{j}$$

#### 2.3.4. Stepwise Linear Regression Model

The standardized data of each principal component and various types of land use areas were used to perform linear regression analysis and determine the main driving forces affecting the various types of forest changes [75,76]. The linear regression model is as follows:

(9) $$Y=\beta_{0}+\beta_{1}T_{1}+\beta_{2}T_{2}+\ldots +\beta_{n}T_{i}$$

where $\beta_{0},\beta_{1},\ldots ,\beta_{n}$ refer to the standardized data of various types of land use, and $T_{1},T_{2},\ldots ,T_{i}$ denote the values of each principal component.

## 3. Results

### 3.1. Forests Carbon Stocks

**Figure 3:**
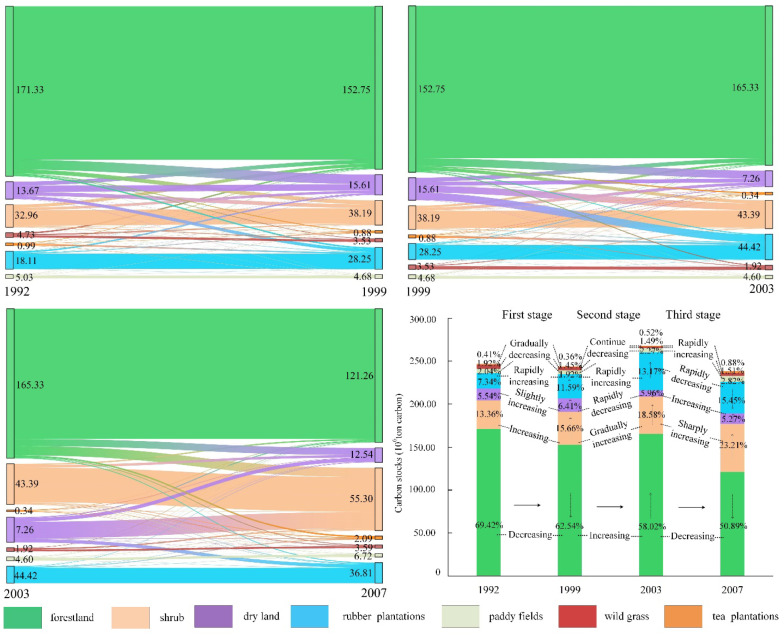
Changes in carbon stocks in Xishuangbanna.

**Figure 4:**
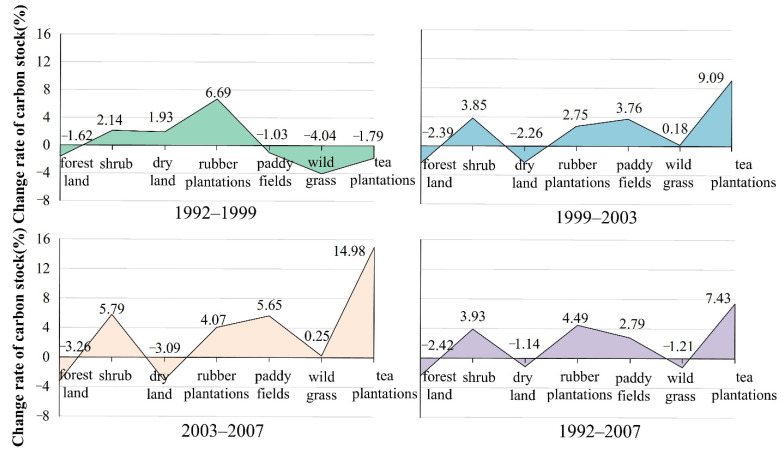
Change rate of carbon stocks in Xishuangbanna.

The carbon stocks in Xishuangbanna are shown in Figure 3 and Figure 4. A continuous decrease in the forest carbon stocks can be observed for the period. The proportion of carbon stocks decreased from 69.42% to 50.89% at an average annual growth rate of 2.42%, especially during 2003 to 2007, at an average annual growth rate of 3.26%. In addition, the carbon stocks in wild grass also showed a downward trend at an average annual growth rate of 1.21%.

The carbon stocks in rubber plantations increased by more than double, from 7.34% in 1992 to 15.45% in 2007, resulting in an average annual growth rate of 4.49%. However, the annual growth rate of rubber plantations showed a slowing trend. The carbon stocks in tea plantations showed an overall upward trend as its proportion increased from 0.41% in 1992 to 0.88% in 2007. Thus, it increased at an average annual rate of 7.43%, faster than the case for rubber. Notably, from 2003 to 2007, the annual change rate of the carbon stocks in tea plantations was as high as 14.98%, indicating the continued growth trend. From 1992 to 2003, the proportion of forestland stocks converted to rubber plantations fell sharply, followed by a rapid rise, almost reaching the original level. Simultaneously, the proportion of forestland stocks converted to tea gardens increased. On the whole, the forests stocks were mainly converted into rubber plantations, whereas the proportion converted to tea gardens was small, albeit showing a rapidly increasing trend. This analysis indicates that the growth rate of rubber plantations has slowed down and that while the area diverted for tea gardens is small, its growth has been quite rapid. Thus, it is likely that more carbon stocks in forestland will be diverted to tea plantations in the future.

The carbon stocks in shrub land continued to increase; its proportion equaled 13.36% in 1992 and increased to 23.21% by 2007, translating to an average annual growth rate of 3.93%. This growth trend continues to increase. The increase of carbon stocks in 2003–2007 was 1.56 times those in 1999–2003 and 1.32 times those in 1992–1999. On the whole, the forest degradation in the study area is becoming increasingly severe.

Combining the abovementioned information on deforestation, forestland transformation, and forest degradation from 1992 to 2007, the carbon stocks in forestland continued to reduce at an accelerated pace, and the deforestation phenomenon was the most severe during the period 2003 to 2007. Most of the existing forestland was cut down and developed into cultivated land, transformed into rubber plantations, or replaced by secondary shrub forests and woodland pastures due to the degradation of the forest ecosystem.

### 3.2. Results of the Principal Component Analysis

As explained previously, although the socio-economic driving forces of forest change were divided into five categories, different degrees of correlation exist between indicators belonging to the same category and those belonging to other categories. In other words, direct analysis of the data of the 26 indicators of the five major socio-economic driving factors is too complicated, and it may not be possible to obtain correct results due to the problems posed by multi-collinearity. Thus, in this study, the 26 indicators of the five major social and economic driving factors were first analyzed to extract independent comprehensive indicators. Then, the standardized socio-economic data from 1992 to 2007 were analyzed to obtain the characteristic values, contribution rates, and cumulative contribution rates of each principal component (Table 3) and the principal component loading matrix (Table 4).

As per Table 3, the eigenvalues of the first, second, third, and fourth principal components are greater than 1, and their cumulative contribution rate reaches 95.14%. These values met the analysis requirements. Therefore, only the first, second, third, and fourth principal components were selected for the study as they comprehensively reflected the status of Xishuangbanna’s social and economic drivers.

The following important points can be drawn from Table 4:

V1 to V16, V18, V19, V22, V23, V25, and V26 are significantly positively correlated to the first principal component. These indicators reflect the importance of economic development, living standards, agricultural technology, population, and agricultural development and structure. Thus, the first principal component is associated with the majority of the socio-economic development indicators.

V17, V20, and V24 are significantly positively correlated to the second principal component, reflecting the importance of the agricultural development level. Thus, the second principal component is associated with the agricultural development level.

V10 and V15 are highly negatively correlated with the third principal component, reflecting the adverse effects of living standards and economic development on forests. Thus, the third principal component is associated with the agricultural development level and economic development.

The fourth principal component is highly correlated with V21, reflecting the importance of afforestation. Thus, the fourth principal component is associated with policy interventions.

### 3.3. Results of the Stepwise Linear Regression Model

**Figure 5:**
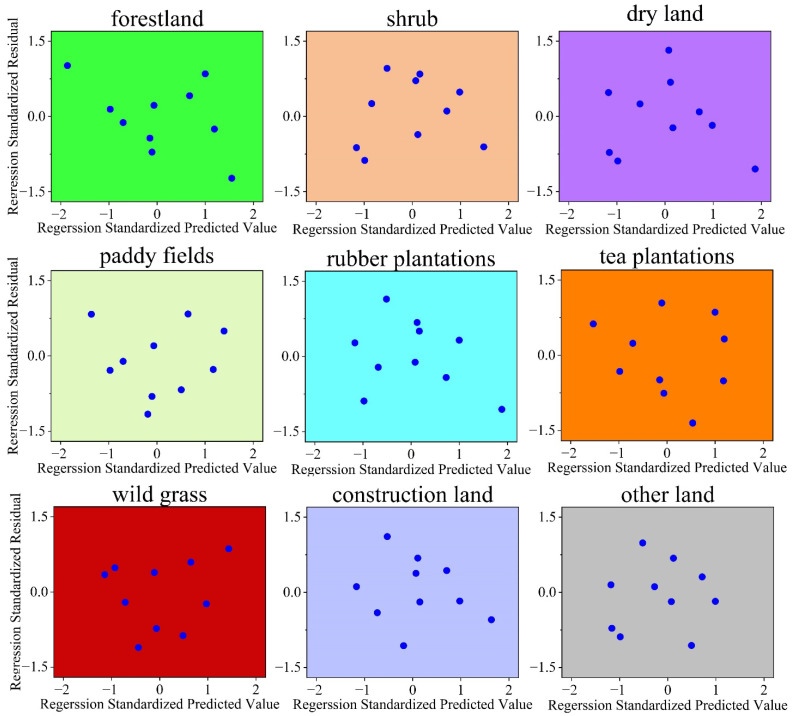
The results of regression standardized residual versus regression standardized predicted values are shown in the figure above. It can be seen that the residuals of each regression equation are randomly distributed around the interval of (−2, 2), and thus the residuals meet normality and independence. As a result, these stepwise linear regression equation models pass significance inspection.

Each principal component was expressed as a linear combination of each driving index variable using the principal component loading matrix. The value of each principal component was thus obtained. Then, the value of each principal component and various types of land use areas (standardized data) were used to perform a stepwise linear regression analysis to obtain the main driving forces affecting forest change. Table 5 shows the results of the stepwise linear regression analysis and Figure 5 shows residuals versus predicted values.

#### 3.3.1. Forces Driving Deforestation

The regression analysis shows that the area categorized as dry land is not significantly negatively correlated to socio-economic development, policy interventions, and other factors. However, it is significantly positively correlated to agricultural development and structure. Moreover, 20 to 30% of the reduction in the forestland area was converted to cultivated land.

The regression equation for paddy fields shows that this area is not significantly positively correlated with socio-economic development, is negatively correlated with agricultural development and structure, and is significantly positively correlated with policy interventions. This shows that socio-economic development has little impact on paddy fields areas, whereas the opposite is true of policies, which can have important impacts on the protection of farmland and paddy field areas. Furthermore, suitable policies can effectively limit erosion by encouraging the growth of paddy fields and inhibiting deforestation.

The regression equation for wild grass shows that the area covered by wild grass is not significantly negatively correlated with socio-economic development and not significantly positively correlated with policies, but it is significantly positively correlated with agricultural development and structure. The net output value of animal husbandry is the main driving factor in the agricultural development level, indicating that animal husbandry development is conducive to the growth of barren grasslands. Thus, attention should be paid to adjusting the agricultural structure by fostering forestry development, increasing forestry output value, and inhibiting deforestation.

The regression equations for construction land and other land show that these areas are not significantly positively correlated with socio-economic development and agricultural development and structure, but they are significantly negatively correlated with policy interventions. Thus, suitable policies can effectively limit the diversion of forestland to the expansion of construction land and other land.

Based on the above information, the most effective measures to suppress deforestation involve adjusting the agricultural structure, promoting forestry development, and formulating strict forest protection policies.

#### 3.3.2. Forces Driving Forestland Transformation

It is worth noting that the regression equation for rubber plantations shows that this land area is positively correlated to socio-economic development and agricultural development and structure, but the correlations are not significant. However, the correlation to policy interventions is significantly positive. Rubber is an important strategic material in China, and it was introduced to Xishuangbanna in 1948. After the creation of the People’s Republic of China, the government vigorously supported rubber cultivation and opened several state-owned farms. In 1982, the state government followed the recommendations of the national rural economic policy and allowed the private development of rubber. The local and central governments provided strong policy support. For example, from 1982 to 1983, 1.786 million yuan were devoted to convert farmlands into rubber plantations. The Agricultural Bank of China and the Xishuangbanna Central Branch provided loans worth 2.3 million yuan to support private rubber production in the whole state. Since then, rubber cultivation has developed very rapidly and in catastrophic yearly proportions in Xishuangbanna. Large areas of natural forest were felled and replaced by rubber plantations. In 1998, the Chinese government began to promote natural forest protection projects by restricting the expansion of rubber plantations to some extent. In 2002, Xishuangbanna began to implement projects converting cropland to forestland. As an economic forest species, however, rubber plantations enjoy subsidies. Since 2006, Xishuangbanna Prefecture has provided subsidies for improving rubber varieties, and since 2007, it has completed technical training for 10,000 rubber plantation farmers every year. This shows that rubber cultivation is closely supported by local policies.

The regression equation for tea plantations shows that this land area is not significantly positively correlated with socio-economic development, but a significant negative correlation exists with policy interventions. In other words, restricting the conversion of forestland into tea gardens is only possible by an effective strengthening of policies.

#### 3.3.3. Forces Driving Forest Degradation

The regression equation for shrubs shows that this area is not significantly positively correlated with socio-economic development and not significantly negatively correlated with agricultural development and structure. However, it is significantly negatively correlated with policy interventions. Forest degradation is not only an important evaluating aspect of sustainable forest management but also a comprehensive reflection of a series of environmental, economic, and ecological issues. The shrub area increased at the cost of decreasing forest land coverage in the study area. From 1992 to 2007, the shrub area increased by more than twofold, which is the embodiment of the decline of forest quality in the forest structure [77]. Farmers in Xishuangbanna tended to replace natural forests with shrub to increase economic income. Approximately half the reduction in forested land is attributable to conversion to shrubs, leading to serious degradation of the forest ecosystem. Thus, strengthening protection via policy interventions can effectively suppress forest degradation.

## 4. Discussion

The following was discussed based on the abovementioned analyses:

(1) Previous studies [78,79,80] indicated that rubber plantations and tea plantations replaced Xishangbanna’s most biodiverse native forests due to local priority policies. National policies to protect native forests from clearance and overexploitation, or to encourage reforestation, are interpreted at a local level by county and village officials. Local governments, whose major objectives are improving the local economy and eradicating poverty, are promoting rubber plantations and tea plantations as a means to diversify smallholder incomes. Results from our study support such remarks. The regression equation for rubber plantations shows that the correlation to policy interventions is significantly positive. Furthermore, the model also shows that restricting the conversion of forestland into tea gardens is only possible by an effective strengthening of policies.
(2) REDD+ provides a useful mechanism for forest-related carbon sequestration and, thus, can contribute to controlling rising CO_2_ levels and help mitigate global warming. As all of REDD’s current programs have been implemented in countries in or near the tropics, the Xishangbanna region plays an important role for China involving REDD+. In this region, China can make contributions to REDD+ through stopping deforestation and forest degradation to reduce emissions.
(3) Direct drivers of deforestation and forest degradation refer human activities or immediate actions that directly impact forest cover and loss of carbon. The most important direct driver is agriculture expansion, which has been identified as the key driver of deforestation in the tropics in the 1980s and 1990s [81,82,83]. In Xishangbanna, our study shows that 20 to 30% of the reduction in forestland was attributable to conversion to cultivated land. Moreover, due to the huge economic benefits of rubber plantations and the national policy support provided to them, the area under these plantations continues to grow at an annual rate of 6.88% [84,85,86].
(4) The direct drivers are considered separately for deforestation and forest degradation [87]. As mentioned above, agriculture expansion is considered as the direct driver of deforestation in Xishuangbanna, while activities such as logging, uncontrolled fires, livestock grazing in forests, and fuel wood collection and charcoal production are considered to be drivers of forest degradation. Our study reveals that in each set of analyzed years, 40% to 50% of the reduction in forested land was attributed to conversion to shrub and grassland due to the degradation of the forest ecosystem.
(5) Rademaekers et al. [88] indicate that poor governance, corruption, low capacity of public forestry agencies, land tenure uncertainties, and inadequate natural resource planning and monitoring can be important underlying factors for deforestation and forest degradation. This is especially true in Xishuangbanna. Imperfect forestland protection policies have led to problems in the forestry management system, and thus, the forestland cannot be fully protected [89]. For example, rubber is an economic forest species, and the activities concerning these plantations are classified as returning farmland to forestland. However, the conversion of forestland into rubber plantations has degraded the forest ecosystem to a certain extent. In addition, the lack of clear property rights associated with forestry resources serve as major barriers in forestry management in Xishuangbanna [90]. For instance, rampant smuggling of timber due to collusion between the staff of the forestry department and illegal elements has been reported [91]. These aspects point to the failure to fully protect forested land.
(6) Gregersen et al. [92] indicate that opportunity costs can be a starting point to determine appropriate levels of funding to stem driver activity. Ecofys also indicates that opportunity costs approach should complement efforts to address underlying drivers and enabling factors, including strengthening governance or bundling incentives [93]. In Xishangbanna, in an effort to protect forest resources, a large number of people have been forced to return farmland for reconversion to forestland. This has affected farmers’ incomes negatively, and thus, their enthusiasm for protecting forestry has decreased [94,95,96]. In view of this, the government should establish an effective connection mechanism between farmers returning farmland to forests and the market to resolve the ironic contradictions that farmers typically face in this regard. The government should also adopt a public expenditure policy to economically promote such conversions while expanding employment and raising farmers’ incomes.
(7) Recently, restoration efforts in Xishuangbanna are increasingly being used to combat tropical rainforests loss. The Xishuangbanna government has implemented different ecological protection policies and measures at different stages of development. In 2007, the Forestry Development Plan of Xishuangbanna during the 11th Five-Year Plan was specially prepared according to national forestry laws and regulations. On 29 June 2018, the People’s Government of Yunnan Province enacted the Ecological Protection Red Line of Yunnan Province, which included three types of red lines, namely, biodiversity maintenance, water conservation, and water and soil conservation in 11 sub-regions. Among them, the ecological protection red line of tropical forest biodiversity maintenance at the southern border covered five prefectures and cities, including Xishuangbanna. In 2021, the People’s Government of Yunnan Province issued the Opinions on the Comprehensive Implementation of the Forest Chief System, which required strengthening the protection of ecological resources, accelerating the ecological restoration of forest and grassland resources, and deepening the reform in forest and grassland planning [97]. With the implementation of ecological protection policies in Xishuangbanna in recent years, the forest coverage of the whole region has increased to 81.34% in 2020, while the ecological environment has also been greatly improved [98].
(8) Landsat-5 and Landsat-7 were operated from 1984 to 2013 and from 1999 to now, respectively. Due to the availability, accessibility and quality of Landsat data, satellite images of Landsat-5/7 TM and ETM+ from 1992, 1999, 2003, and 2007 were used to obtain information on land use changes. At present, the gradual aging of the sensor characteristics and the satellite’s orbit positioning accuracy may lead to a certain degree of decline in the radiation accuracy and geometric positioning accuracy for the current imageries of Landsat-5 and Landsat-7 [99]. The latest in-orbit Landsat-9 is equipped with the second-generation Land Imager (OLI-2) and the second-generation Thermal Infrared Imager (TIRS-2), which have improved the radiation resolution and SNR (signal-to-noise ratio) significantly [100,101,102]. Furthermore, Landsat-9 and Landsat-8 can be used for collaborative and complementary observation. Such temporal resolution of 8d can effectively promote the ecologically monitoring capability [103]. Therefore, in future studies Landsat data with higher temporal resolution and higher spatial resolution can contribute to improve the accuracy of the model in Xishuangbanna.

## 5. Conclusions

In summary, the identification and analysis of the forces driving forest change in Xishuangbanna show that it is crucial to apply the REDD+ framework to stop deforestation in the area and to restore the degraded forests. As tropical forests continue to be cut down and the area converted to other land use types, the carbon storage of vegetation in the Xishuangbanna region has also shown a downward trend, with its distribution pattern changing from a random discrete distribution to an aggregated one. The core forest area has decreased over time; the number of patches has decreased, as has the distance between them. Significant changes have taken place in the forest landscape structure, and the tendency for landscape fragmentation is evident [104]. Increased soil erosion causes further degradation of the land, resulting in the sedimentation of rivers and lakes, in turn reducing the ability of forests and soil to control floods [105]. These aspects have seriously affected the service functions of forest ecosystems, including that in the study area. Application of the REDD+ framework will not only help increase the carbon storage level but will also bring many ecological benefits, ultimately enhancing China’s ability to respond to climate change.

## Figures and Tables

**Table 1:** Selection of socio-economic factors driving forest change in Xishuangbanna.

Number	Type	Driving Factors (Units)
I	Population	V1: Total population at the end of the year (ten-thousand persons) V2: Non-agricultural population (ten-thousand persons) V3: Rural labor force (persons) V4: Number of employees in agriculture, forestry, animal husbandry, and fisheries (persons)
II	Economic development	V5: GDP * (ten-thousand Yuan) V6: GDP of the primary industries (ten-thousand Yuan) V7: Revenue (ten-thousand Yuan) V8: Fiscal expenditure (ten-thousand Yuan) V9: Total retail sales of social goods (ten-thousand Yuan) V10: Investment in fixed assets (ten-thousand Yuan) V11: Highway mileage (km)
III	Living standards	V12: Year-end balance of savings deposits of urban and rural residents (ten-thousand Yuan) V13: Per capita net income of farmers (Yuan/person)
IV	Agricultural development level	V14: Total agricultural net output value (ten-thousand Yuan) V15: Net output value of planting industry (ten-thousand Yuan) V16: Forestry net output value (ten-thousand Yuan) V17: Animal husbandry net output value (ten-thousand Yuan) V18: Net fishery output value (ten-thousand Yuan) V19: Gross agricultural output (ten-thousand Yuan) V20: Sown area of main crops ** (ha) V21: Returning farmland to forests (ten-thousand mu) V22: Rubber production (t) V23: Tea production (100 kg) V24: Food production (t)
V	Agricultural technological progress	V25: Rural electricity consumption (ten thousand kWh) V26: Fertilizer application (scalar t)

* GDP: Gross domestic product. ** main crops: cereal, beans, and tubers. Source: Yunnan Statistical Yearbook [64].

**Table 2:** Parameters in carbon stocks evaluation (ton C/ha).

Land Use Type	Soil Carbon Density	Vegetation Carbon Density	Total Carbon Density	Source of Data
Forestland	99.57	45.30211	144.8721	ZhangXiuyu, Li Hongmei et al. [66,67]
Shrub	109.2	9.534	118.734	ZhangXiuyu, Li Hongmei et al. [66,67]
Tea plantations	20.662	12.1768	32.8388	Xiao Ziwei [68]
Rubber plantations	104.7	66.79645	171.4965	Pang Jiaping, ShaLiqinget al. [69,70]
wild grassland	60.6	4.935	65.535	Zhang Xiuyu, XieXianli et al. [66,71]
paddy fields	103	0	103	ShaLiqinget al. [70]
dry land	61.9	0	61.9	XieXianli et al. [71]

**Table 3:** Eigenvalues and principal component contribution rates.

Composition	Eigenvalue	Contribution Rate (%)	Cumulative Contribution Rate (%)
1	18.940	72.845	72.845
2	2.881	11.081	83.925
3	1.828	7.030	90.955
4	1.087	4.181	95.136

Note: To extract the number of principal components, the first *n* principal components whose feature values corresponding to the principal component is greater than 1 was considered.

**Table 4:** Principal component loading matrix.

Socio-Economic Driving Factors	Composition				Socio-Economic Driving Factors	Composition			
	1	2	3	4		1	2	3	4
V1	0.881	0.055	−0.437	−0.114	V14	0.976	−0.026	0.018	0.125
V2	0.851	−0.192	0.264	−0.24	V15	0.719	0.075	−0.66	−0.144
V3	0.939	−0.288	0.16	0.004	V16	0.938	−0.24	−0.046	0.159
V4	0.931	−0.318	0.131	−0.01	V17	0.595	0.72	0.227	−0.137
V5	0.987	−0.059	0.075	−0.004	V18	0.83	0.345	0.142	−0.262
V6	0.975	0.107	−0.061	0.115	V19	0.907	0.031	−0.406	−0.046
V7	0.742	0.245	0.377	0.103	V20	0.005	0.929	0.262	0.105
V8	0.966	−0.197	0.079	−0.06	V21	0.663	0.124	−0.202	0.689
V9	0.991	−0.051	0.056	−0.002	V22	0.727	0.288	0.119	0.466
V10	0.819	0.125	−0.519	−0.061	V23	0.948	0.17	−0.184	0.026
V11	0.803	−0.415	0.377	−0.088	V24	0.449	0.748	−0.075	−0.309
V12	0.985	−0.135	0.035	0.01	V25	0.957	−0.187	0.163	−0.027
V13	0.939	0.093	0.295	−0.037	V26	0.964	−0.197	−0.013	−0.158

**Table 5:** Results of the stepwise linear regression analysis of factors driving land use change in Xishuangbanna and their residuals versus predicted values.

Land Use Types	Regression Equation	*R* ^2^	VIF	*p*-Value	RMSE
forestland	$Y=1.007-0.57X_{1}+0.002X_{2}+0.223X_{4}$	0.902	1.000	0.015	0.002
shrub	$Y=0.02+0.06X_{1}-0.096X_{2}-0.302X_{4}$	0.811	1.000	0.024	0.001
dry land	$Y=0.26-0.042X_{1}+0.557X_{2}-0.028X_{4}$	0.914	1.000	0.019	0.003
paddy fields	$Y=0.169+0.061X_{1}-0.372X_{2}+0.636X_{4}$	0.801	1.000	0.035	0.002
rubber plantations	$Y=-0.078+0.053X_{1}+0.103X_{2}+0.307X_{4}$	0.852	1.000	0.009	0.002
tea plantations	$Y=0.213+0.061X_{1}-0.291X_{2}-0.876X_{4}$	0.831	1.000	0.041	0.001
wild grassland	$Y=1.123-0.03X_{1}-0.482X_{2}+0.052X_{4}$	0.752	1.000	0.009	0.002
construction land	$Y=0.016+0.054X_{1}+0.051X_{2}-0.603X_{4}$	0.803	1.000	0.017	0.003
other land	$Y=0.112+0.042X_{1}+0.243X_{2}-1.276X_{4}$	0.821	1.000	0.035	0.002

Note: *X*_1_, *X*_2_, and *X*_4_ are the first principal component (related to socio-economic development indicators), the second principal component (related to the agricultural development level), and the fourth principal component (related to policy interventions). *Y* is the land use change area. VIF: variance inflation factor. RMSE: root mean-square error.

## Data Availability

The datasets generated and/or analyzed during the current study are available from the corresponding author on reasonable request.
